# The relationship between pedal force application technique and the ability to perform supramaximal pedaling cadences

**DOI:** 10.3389/fspor.2022.958827

**Published:** 2022-08-16

**Authors:** Yuta Yamaguchi, Mitsuo Otsuka, Kohei Watanabe, Naoki Wada, Tetsunari Nishiyama

**Affiliations:** ^1^Faculty of Sport Science, Nippon Sport Science University, Setagaya, Japan; ^2^Laboratory of Neuromuscular Biomechanics, School of Health and Sport Sciences, Toyota, Japan

**Keywords:** index of force effectiveness, high pedaling cadence, steady-state pedaling, technique, cycling

## Abstract

This study aimed to examine the relationship between the pedal force application technique under a specific competitive condition and the ability to perform steady-state pedaling at a supramaximal cadence during a special pedaling test. A total of 15 competitive male cyclists and 13 active, healthy men (novice cyclists, hereafter, novices) performed the pedaling technique test. The test imitated a road cycling competition condition (80% VO2 peak and a cadence of 90 rpm). Additionally, they performed a supramaximal cadence test that evaluated the ability to perform steady-state pedaling for an ultra-high cadence (range of 160–220 rpm) of 30 s stably with a 0.1 kgf. For the pedaling technique test, kinetic data were obtained by the pedal-shaped force platform at 1,000 Hz, and the pedaling technique was determined by the index of force effectiveness (IFE). For the supramaximal cadence test, kinematic data were obtained using a motion capture system at 200 Hz. The supramaximal pedaling cadence (C_max_) was determined by measuring exercise time and targeted pedaling cadence. The IFE was 48.0 ± 9.7% in cyclists and 32.0 ± 5.9% in novices. The C_max_ was 215.5 ± 8.8 rpm in cyclists and 192.2 ± 13.0 rpm in novices. These values were significantly higher for cyclists than for novices. C_max_ was moderately correlated with IFE (*r* = 0.64). No significant correlation was observed between C_max_ and IFE for cyclists only; in contrast, a moderate correlation was observed between these parameters for novices only (*r* = 0.67). In conclusion, the pedal force application technique under a specific competitive condition is related to the ability to perform steady-state pedaling for supramaximal cadence during the test. Therefore, C_max_ may be able to explain pedal force application techniques without the need for expensive devices for novices.

## Introduction

Crank power production is a crucial determinant of cyclists' speed and track performance (Dorel et al., 2005). The crank power can be calculated as the product of the crank torque and angular velocity (pedaling cadence). Crank torque is related to muscle force factors, such as the cross-sectional area of the muscle, type of muscle fiber (McCartney et al., 1983), and coordination in the lower limbs (Korff et al., 2007). Although previous studies have investigated the determinants of optical pedaling cadence (Ansley and Cangley, 2009), no study has reported the determinants of higher pedaling cadence to the best of our knowledge.

The relationship between crank power and pedaling cadence during a sprint can be determined using second-order polynomial regression (Samozino et al., 2007). The peak crank power attains at ~120 rpm, whereas it decreases at higher pedaling cadences. The decreased power output at higher pedaling cadences may be due to a remarkable reduction in the mean value of crank torque for one crank cycle (Samozino et al., 2007). This reduced crank torque results from a decreased positive crank torque during the downstroke phase (Samozino et al., 2007) and an increased negative crank torque during the upstroke phase (Samozino et al., 2007; Dorel et al., 2010). At higher pedaling cadences, the force produced by the lower-limb muscles, which is related to crank power production, appears at a more delayed crank angle relative to the optimal angle for maximizing crank torque (Samozino et al., 2007). The crank torque (or pedal force) generation at the optimal crank angle is attributed to smooth lower-limb muscle coordination (Samozino et al., 2007; Blake and Wakeling, 2015).

The cyclist appropriately adapts the muscles' activation/deactivation point and duration according to pedaling cadence (Chapman et al., 2008). Smooth lower-limb muscle coordination can be considered a refined pedaling skill. It has been reported that novices with unrefined skills are more likely to employ the co-activation of lower-limb muscles with increasing cadence (Chapman et al., 2008). Therefore, at higher pedaling cadences, it seems to be important to improve the lower-limb muscle coordination; how to apply the pedaling force, which is necessary to activate the lower-limb extensor muscles at the optimal crank angle and deactivate those muscles with the other angles (So et al., 2005; Chapman et al., 2008). This would lead to the continuous generation of higher crank power throughout the entire crank rotation.

The pedal force application technique is the ability to control the pedaling force perpendicular to the crank (Korff et al., 2007). This technique has been evaluated using the index of force effectiveness (IFE) (Coyle et al., 1991; Zameziati et al., 2006; Candotti et al., 2007; Korff et al., 2007). Several previous studies have reported that IFE at the normal pedaling cadence is related to competition levels, cycling economy (Candotti et al., 2007), and cycling efficiency (Zameziati et al., 2006). However, it seems financially and technically difficult for cyclists to assess their pedaling technique because expensive facilities and bioengineering skills are required for these measurements.

The lower accuracy of normal-speed motion is more prominent at higher speeds in novices than experienced athletes (García et al., 2013). Adaptation to increasing pedaling cadence with better lower-limb muscle coordination is more often observed in cyclists than in novices (Chapman et al., 2008). This suggests that the interindividual pedaling technique might be more pronounced at a higher pedaling cadence. Thus, a pedal force application technique at a normal cadence would relate to the ability to perform stable and repetitive exercises at a higher specific pedaling cadence.

Therefore, this study investigated the relationship between pedaling technique in a specific competitive condition and the ability to perform steady-state pedaling during supramaximal cadence. The test was developed for the first time in our study. We hypothesized that the pedaling technique at a normal pedaling cadence would be related to the ability to perform steady-state pedaling at an ultra-high pedaling cadence during the supramaximal test. These results may be a more convenient method for athletes to evaluate pedaling techniques easily, and coaches could help improve training and cycling performance.

## Materials and methods

### Participants

A total of 28 active and healthy men (age, 22.3 ± 3.7 years; height, 1.73 ± 0.05 m; and mass, 68.4 ± 8.1 kg) volunteered for the study. The participants included 15 cyclists (20.0 ± 0.9 years; 1.71 ± 0.04 m; and 67.4 ± 8.3 kg; with cycling experience of more than 4 years, range: 4–7 years) and 13 novices (25.0 ± 3.9 years; 1.75 ± 0.06 m; and 69.6 ± 7.9 kg). In the last 2 years, none of the novices had suffered from injuries, which would affect the present pedaling experiments (De Bernardo et al., 2012). They received an explanation regarding the study's purpose and procedures and signed an informed consent form for inclusion before participating. The study was conducted in accordance with the Declaration of Helsinki, and the protocol was approved by the Ethics Committee of Nippon Sport Science University (017-H072).

### Protocol

Three experiments were conducted in this study. To measure peak oxygen uptake (VO2peak), the participants performed an incremental pedaling exercise test during the first visit. The participants completed a pedaling technique test on the second visit, 1 day or more after the first visit. Finally, on the third visit, 1 day or more after the second visit, they performed a developed supramaximal cadence test. During the three cycling tests, cyclists were asked to maintain the same position as in the cycling competitions. The handle position was a drop handle posture to ensure that the trunk angle was 12°, measured between the greater trochanter and acromion line with respect to the horizontal plane (Fintelman et al., 2015). For novices, saddle height (distance from the saddle surface to the pedal axis) was determined based on the participant's leg length (distance from the greater trochanter to the sole). These tests used a strap pedal that secured the foot and pedals with a leather belt, and the participants wore conventional athletic shoes.

### Incremental pedaling exercise test

VO2peak was obtained using an incremental exercise test on an electronically braked cycle ergometer (PowerMAX V3, Combi, Tokyo, Japan) adapted with drop handlebars and a racing saddle to determine the pedaling load during the next pedaling technique test. The incremental exercise test measured oxygen uptake (VO2) using a metabolic measurement system (AE-310S; Minato Medical Science Company, Kanagawa, Japan). The VO2 and carbon dioxide produced were monitored every 20 s at the end of each step for 3 min. VO2peak was determined from the average of the highest values obtained for 10–20 s during the test. The incremental pedaling exercise test was finalized based on at least three of the following five criteria: (1) VO2 reached a steady state despite increasing workloads; (2) a predicted maximal heart rate [HRmax (bpm) = 220 (bpm) – age (y)] was achieved at 90%; (3) participants voluntarily quit exercise due to exhaustion; (4) participants were unable to maintain the target cadence; and (5) the Borg rating of perceived exertion reached more than 17. To ensure the accuracy of the VO2peak measurement, a threshold of −2 rpm was set for the target pedaling cadence in this study (cyclists: below 88 rpm, novices: below 58 rpm). The peak power output was determined when VO2peak was achieved. The protocol for cyclists and novices was followed with an initial load of 1.0 kgf, with increments of 0.5 kgf every 3 min. The pedaling cadence for cyclists and novices during the test was 90 and 60 rpm, respectively, and they were instructed to maintain the pedaling cadence within ± 1 rpm. The set cadence was based on the most physiologically efficient cadence from previous studies (Takaishi et al., 1998; Neptune and Herzog, 1999). The pedaling power at 80%VO2peak was determined using first-order linear regression analysis for each participant (dependent variable: pedaling power; independent variable: VO2).

### Pedaling technique test

To evaluate the pedaling technique, the participants performed a pedaling technique test at a normal cadence with the same ergometer used in the incremental pedaling exercise test. Competitive cycling often occurs at 80%VO2peak and 70–100 rpm cadence (Hagberg et al., 1981; Coyle et al., 1991; Takaishi et al., 1996; Faria et al., 2005). Therefore, our study conducted the pedaling technique for 1 min at 80%VO2peak and 90 rpm cadence in a competitive cycling position. This test began with a warmup session comprising 5 min of pedaling at a pedaling load of 1.0 kgf and a cadence of 90 rpm.

### Supramaximal cadence test

**Figure 1:**
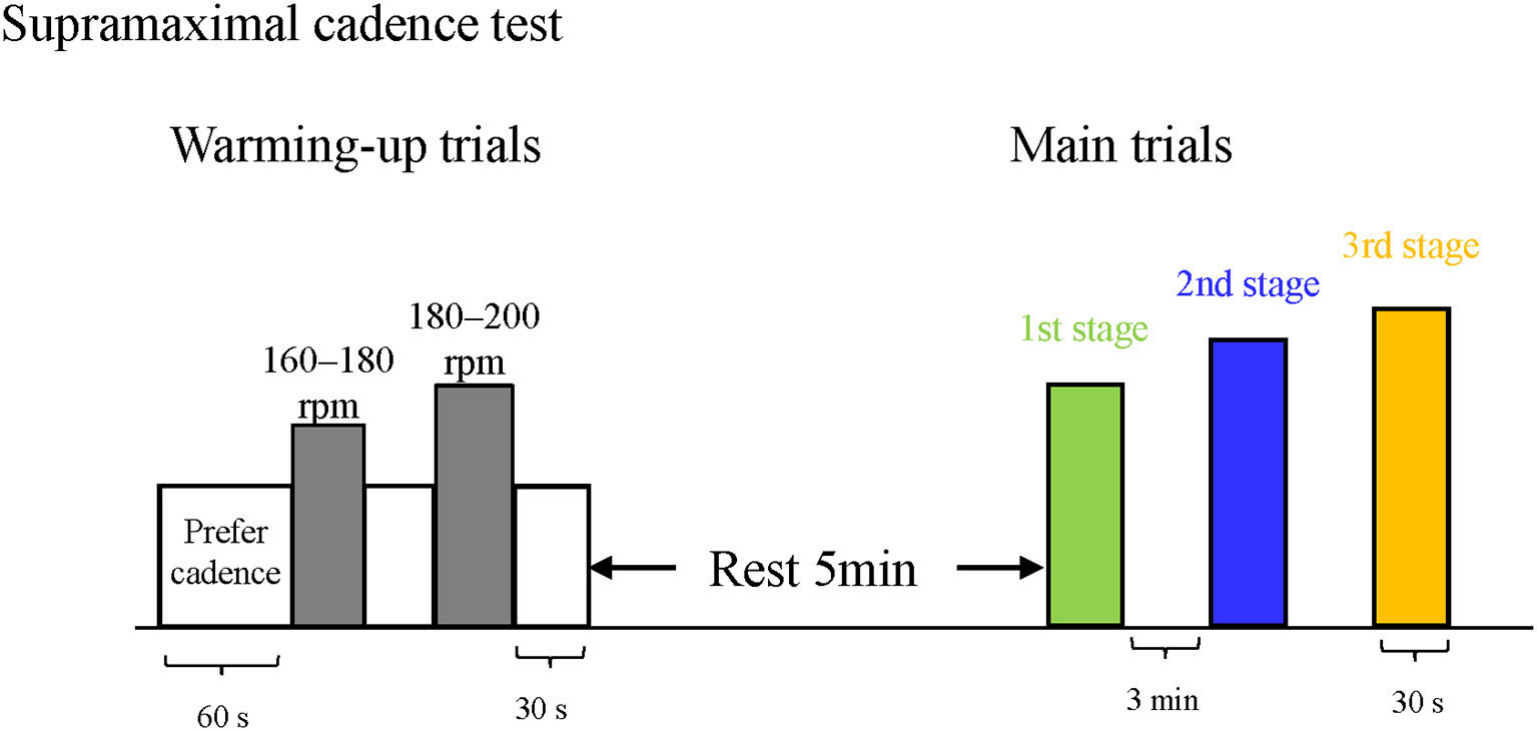
The basic protocol for the supramaximal cadence test. Participants performed standardized warmup training for 3 min. They then performed the supramaximal cadence test, which consisted of three (for this figure) or four main intermittent cadence trials after a 5-min rest period.

After the pedaling technique test, the participants performed a supramaximal cadence test, which we originally developed originally for our study, to quantify and evaluate the cycling ability to maintain the cadence set as high as possible with a low load (0.1 kgf). This test comprised three main intermittent cadence trials to minimize the effect of fatigue after five warmup trials (Figure 1).

First, for the warmup session, the participants performed standardized warmup pedaling for 60 s at the preferred cadence, the first high-cadence pedaling for 15 s, a preferred cadence pedaling for 45 s, the second high-cadence pedaling for 15 s, and a preferred pedaling for 45 s. In the first high-cadence pedaling, the participants were asked whether they could maintain the determined cadence (180–200 rpm for cyclists and 160–180 rpm for novices) even after the pedaling duration was lengthened from 15 to 30 s. If possible, the next cadence during the second high-cadence pedaling was increased by 10–20 rpm.

After sufficient rest for 5 min following the warmup trial, three main trials of intermittent pedaling were conducted. The cadence in the first stage was selected as 190–210 rpm for cyclists and 170–190 rpm for novices, referring to the cadence at the second high-cadence pedaling in the warmup trial. When the participants maintained the target cadence for 30 s, it was increased by 10–20 rpm, and the exercise was performed again after a rest period of 3 min or more. These steps were repeated until the participants could not maintain the target cadence for 30 s (the final stage was the third stage for most participants). The criteria for defining unsustainability were that the participants could not maintain the current rate and that the pedaling cadence was 3 rpm lower than the target cadence for more than 5 s. The pedaling cadence during the supramaximal cadence test was continuously obtained using the SRM system (Schoberer Rad Messetechnik, Jülich, Germany), and it was monitored by both the participants and the tester.

### Data measurement and process

For the pedaling technique test, two-dimensional displacements of all reflective markers in the sagittal plane were sampled at 200 Hz using a three-dimensional motion capture system (MX13, Vicon Motion Systems, Oxfordshire, UK). A 14-mm reflective marker was placed outside the right pedal spindle. For the supramaximal cadence test, two-dimensional displacements of all the reflective markers in the sagittal plane were sampled using the same method as that used for the pedaling technique test.

Kinematic data were filtered using a fourth-order zero-lag low-pass Butterworth filter with a cutoff frequency of 8 Hz (Martin and Brown, 2009). After the filtering procedure, the crank angle was calculated using the reflective marker positions of the pedal spindle and the crank axis. The crank angle was calculated from the crank axis to the pedal axis by using the inverse tangent of the crank displacement vector from the crank axis to the pedal axis. The crank angular velocity was calculated using the time derivative regarding the crank angle and was used as the pedaling cadence. Kinetic data were obtained using a pedal-shaped force platform (PZB0004, Kistler, MI, USA) at 1,000 Hz, and the crank torque was calculated using the pedal force perpendicular to the crank arm and crank length (0.170 m).

During the pedaling technique test, IFE was used to evaluate the pedaling techniques. When the time-series pedaling cadence did not change significantly, kinetic data for 10 consecutive cycles were used for further analysis. IFE was calculated using the following equation:


(1) $$IFE=100\times \frac{\int_{0}^{2\pi }EF\left(\theta \right)}{\int_{0}^{2\pi }RF\left(\theta \right)}$$

where θ is the crank angle, EF is the effective force for the crank rotation, and RF is the resultant force applied to the pedal.

The highest pedaling cadence among the three stages, stably maintained for 30 s (C_max_), was used for further analysis in the supramaximal cadence test. If the participant could not maintain the targeted pedaling cadence for 30 s in the conducted stage, C_max_ was indirectly evaluated considering the exercise time for which the participants could continue to maintain the targeted cadence.


(2) $$C_{\max}=C_{n-1}+(C_{n}-C_{n-1})\times (t_{exer}÷t_{total})\;$$

where n is the stage number, Cn is the targeted pedaling cadence at the n stage, and t_exer_ is the exercise time for which the participant could continue to maintain the targeted Cn (maximal time: 30 s); t_total_ is the total exercise time in the stage (30 s). The mean values of the instantaneous pedaling cadences in 10 consecutive cycles (when the time-series pedaling cadence did not change significantly) were calculated to observe the pedaling cadence profile. All data processing was performed using MATLAB (R2020a version 9.8.0.1451342, MathWorks, Inc., Natick, MA, USA).

### Statistical analysis

Mean values, standard deviations, and coefficients of variation (CV) were used to present variables for the group data. Spearman's correlation coefficient was used to evaluate the relationship between IFE and C_max_. The significance level was set at *p* < 0.05. The magnitude of the relationship between test measures was interpreted based on Cohen's classification scheme (correlation coefficients < 0.5) and was considered to be small, correlation coefficients between 0.5 and 0.8 were considered to be moderate, and correlation coefficients > 0.8 were considered to be large (Cohen, 1988). All statistical analyses were performed using Statistical Package for Social Sciences (SPSS 24.0, IBM, NY, USA).

## Results

The overall IFE was 40.5 ± 11.4% (range: 24.1–61.2%; Table 1). The IFE of cyclists was 48.0 ± 9.7% (range: 26.1–61.2%; Table 1) and that of novices was 32.0 ± 5.9% (range: 24.1–42.0%; Table 1).

**Table 1:** Variables in pedaling technique test and supramaximal cadence test.

	**Total (*****n*** = **28)**			**Cyclists (*****n*** = **15)**			**Novices (n** = **13)**		
	**Mean**	**SD**	**CV**	**Mean**	**SD**	**CV**	**Mean**	**SD**	**CV**
IFE (%)	40.5	11.4	28.1%	48.0	9.7	20.2%	32.0	5.9	15.6%
C_max_ (rpm)	204.7	15.9	7.7%	215.5	8.8	4.2%	192.2	13.0	6.8%

SD, standard deviation; CV, coefficient of variation; IFE, index of force effectiveness during the pedaling technique test; C_max_, maximal pedaling cadence during the supramaximal cadence test.

The overall C_max_ was 204.7 ± 15.9 rpm (range: 167.6–226.5 rpm). The C_max_ of cyclists was 215.5 ± 8.8 rpm (range: 203.1–226.5 rpm), and that of novices was 192.2 ± 13.0 rpm (range: 167.6–217.0 rpm). The CVs of IFE and C_max_ are presented in Table 1.

**Figure 2:**
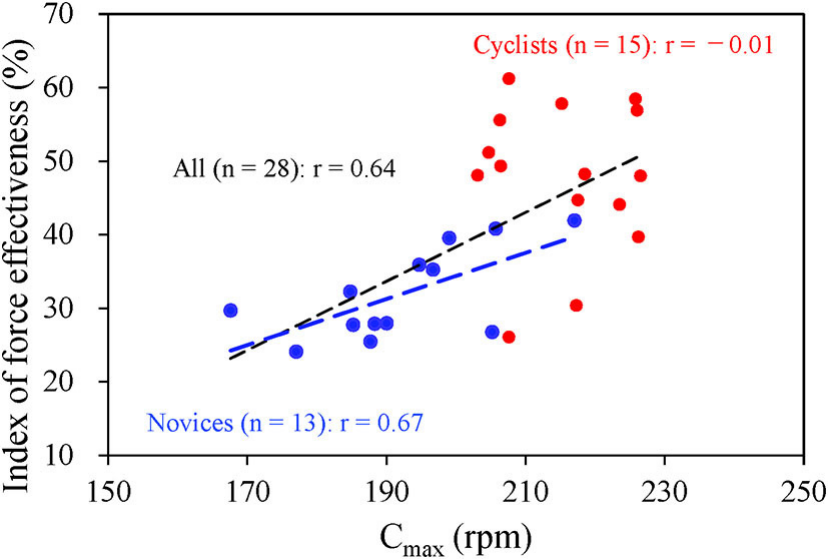
Relationship between the index of force effectiveness during the pedaling technique test and the maximal cadence (C_max_) during the supramaximal cadence test. Red circles denote data from cyclists, blue circles and dashed line indicate data from novices, and a black dashed line indicates data from overall participants.

IFE was moderately correlated with C_max_ (*r* = 0.67, *p* < 0.01; Figure 2). When data from cyclists and novices were separated, no significant correlation was observed between IFE and C_max_ for cyclists alone (*r* = −0.01, *p* = 0.98), whereas a moderate correlation was observed between IFE and C_max_ for novices alone (*r* = 0.64, *p* < 0.01).

## Discussion

This study aimed to investigate the relationship between pedal force application technique during a specific competitive condition and the ability to perform steady-state pedaling at supramaximal pedaling cadence during the supramaximal cadence test, which was newly developed in this study. Therefore, we hypothesized that the pedal force application technique at a normal pedaling cadence would be related to the ability to perform steady-state pedaling at an ultra-high pedaling cadence during the supramaximal cadence test. The key finding of our study was that a positive correlation was observed between IFE at a normal cadence and the C_max_ obtained by the supramaximal cadence test. This finding suggests that the ability to perform steady-state pedaling at the highest possible pedaling cadence can help evaluate the pedaling technique at a normal cadence. Thus, our hypothesis is confirmed.

If a gear is not shifted during cycling, the pedaling load gradually decreases as the cadence and moving speed increase, as previously noted based on the cadence and torque data obtained by an inertial load ergometer (Arsac et al., 1996). In track cycling, using only one gear throughout the race, the pedaling cadence directly contributes to the maximum mechanical power (Dorel et al., 2005) and race record (Craig and Norton, 2001). The maximum pedaling cadence attained over 150 rpm in a track race has been previously reported (Craig and Norton, 2001); however, previous studies have not focused on pedaling behavior under ultra-high cadence. The C_max_ obtained by the supramaximal cadence test was 204.7 ± 15.9 rpm, which is higher than the cadence reported during the steady-state pedaling in previous studies (Neptune and Herzog, 1999: up to 120 rpm; Aasvold et al., 2019: 40–100 rpm). In particular, a C_max_ of 217.0 rpm was not attained by any novice. It was achieved only by cyclists owing to regular training (similarly, for an IFE value over 48.5%). The pedaling intensity during track cycling differed from that during the pedaling technique test. The intensity during the supramaximal test was 0.1 kgf and must be lower than that reported in a previous study (Craig and Norton, 2001). This probably resulted in an ultra-high pedaling cadence (>150 rpm).

Increasing pedaling cadence would have increased the negative crank torque (Neptune and Herzog, 1999; Samozino et al., 2007; Dorel et al., 2010). Samozino et al. (2007) indicated that muscle coordination in the lower limbs was poorly adapted during an over-optimal cadence for maximal output power (e.g., over 120 rpm). Furthermore, the increase in the negative crank torque, which affects the decrease in the crank angular velocity, is known to linearly increase with increases in pedaling cadence (Dorel et al., 2010). Thus, this phenomenon might indicate that each participant could no longer increase the pedaling cadence after attaining maximal pedaling cadence during the supramaximal cadence test.

As expected, a significant positive relationship was observed between IFE and C_max_. Muscle co-activation frequently occurs (Chapman et al., 2008), and muscle coordination tends to be disrupted (Blake and Wakeling, 2015). In the supramaximal cadence test, the response of muscle force generation is likely to be delayed (Samozino et al., 2007; Blake and Wakeling, 2015); therefore, the increase in ineffective force during the entire revolution cycle is considered to decrease the IFE (Blake and Wakeling, 2015). Thus, switching activation and deactivation seems difficult in the lower-limb muscles during high pedaling cadence exercises. In contrast, experienced athletes can enhance their speed by maintaining accuracy (García et al., 2013). These results demonstrate that the IFE of skillful participants is high as they maintain the accuracy of lower-limb muscle coordination despite high pedaling cadences.

However, a significant relationship was not observed between IFE and C_max_ for cyclists alone; therefore, our hypothesis was partially rejected. This may be because the better cyclists could not enhance their C_max_ by more than 230 rpm. The CV of the C_max_ of the cyclists was 4.2%, which was less than approximately one-fifth of the CV of their IFE (20.2%), suggesting that 226.5 rpm, the highest C_max_ of cyclists, is the maximum limit for any cyclist. This value can be regarded as the biological limit of muscle contraction velocity. The C_max_ of many cyclists was pooled at ~225 rpm (*n* = 5, Figure 2). In addition, the competitive level of experienced athletes is not affected by the pedal force application technique and multiple factors, such as muscle fiber type and anaerobic power (Sjödin and Svedenhag, 1985). In accordance with the muscle force–velocity relationship, higher muscular power is contributed by high-speed muscle contraction during the monoarticular motion (Toji et al., 1997). Similarly, the C_max_ of cyclists under high-speed conditions was likely related to the maximal pedaling power output, suggesting long-term training specificity for muscle power generation for greater cyclists. Further research is warranted to investigate why no significant relationship was obtained between IFE and C_max_ of cyclists alone. The effects of other factors on the relationship should be investigated, and the data set of cyclists should be evaluated based on different competitive levels.

In contrast, a moderate correlation coefficient was obtained between the IFE and C_max_ for novices alone, suggesting that the pedal force application technique is a more important determinant of the ability of novices to perform steady-state pedaling with a high cadence. Indeed, the C_max_ was smaller in novices than in cyclists (192.2 ± 13.0 rpm vs. 215.5 ± 8.8 rpm), suggesting a higher trainability to perform a high pedaling cadence during the steady-state test in novices than in cyclists. Therefore, the pedaling technique can be estimated based on the ability to perform steady-state pedaling with a high pedaling cadence during the supramaximal cadence test, particularly for novices. Using general measurement methods to evaluate pedal force application techniques is difficult for athletes and coaches because they require special and expensive equipment. In contrast, the supramaximal cadence test devised in our study could be performed with a participant's bike mounted on a bike trainer stand. Thus, it may be possible for novices and young cyclists to evaluate the effect of technique training by measuring C_max_ longitudinally.

A limitation of this study is that it could not clarify the relationship between the mechanical properties of the pedaling technique test and those of the supramaximal cadence test. This was because crank torque was not obtained with a high pedaling cadence. Perhaps, there are two pedaling strategies for maintaining crank power. One is suppressing the negative crank torque and minimizing the crank torque generated during the entire revolution cycle. The other is to generate a large positive crank torque and cancel the negative crank torque. These pedaling strategies would help to understand better the relationship between the mechanical properties of the pedaling technique test and those of the supramaximal cadence test. These strategies might explain why the relationship was not significantly observed between the IFE and C_max_ for cyclists alone. Future studies should measure crank torque to clarify the torque profile during supramaximal cadence tests.

## Conclusion

This study assessed the relationship between pedaling technique at a normal pedaling cadence and the ability to perform at a high pedaling cadence for 30 s during the supramaximal cadence test. In conclusion, a moderately positive relationship was observed between IFE and C_max_. Therefore, it was indicated that the ability to perform steady-state pedaling at an ultra-high cadence is related to the pedal force application technique. Furthermore, when cyclists and novices were separated, a moderate correlation was observed. Therefore, especially for novices, the pedal force application technique may be easily evaluated using the supramaximal cadence test, which was newly developed in this study.

## Data availability statement

The original contributions presented in the study are included in the article/supplementary materials, further inquiries can be directed to the corresponding author.

## Ethics statement

The studies involving human participants were reviewed and approved by Ethics Committee of Nippon Sport Science University. The patients/participants provided their written informed consent to participate in this study.

## Author contributions

YY and TN: conceived and designed the experiments. YY: performed the experiments and analyzed data. YY, MO, KW, and TN: interpreted results of research. YY, NW, and MO: drafted the manuscript and prepared tables/figures. YY, MO, KW, NW, and TN: edited and critically revised paper and approved the final version of the manuscript. All authors contributed to the article and approved the submitted version.

## Conflict of interest

The authors declare that the research was conducted in the absence of any commercial or financial relationships that could be construed as a potential conflict of interest.

## Publisher's note

All claims expressed in this article are solely those of the authors and do not necessarily represent those of their affiliated organizations, or those of the publisher, the editors and the reviewers. Any product that may be evaluated in this article, or claim that may be made by its manufacturer, is not guaranteed or endorsed by the publisher.
